# Age-related decline in LEPR^+^ hematopoietic stem cell function

**DOI:** 10.1038/s41375-023-01815-1

**Published:** 2023-01-17

**Authors:** Thao Trinh, James Ropa, Scott Cooper, Arafat Aljoufi, Anthony Sinn, Maegan Capitano, Hal E. Broxmeyer, Mark H. Kaplan

**Affiliations:** 1 Department of Microbiology/Immunology, Indiana University School of Medicine, Indianapolis, IN 46202 USA; 2 In Vivo Therapeutics Core, Indiana University School of Medicine, Indianapolis, IN 46202 USA

**Keywords:** Haematopoietic stem cells, Leukopoiesis

## To the Editor:

With the steady growth in the numbers of older individuals in the population worldwide, understanding how aging affects tissue-specific stem cells including the hematopoietic system will provide valuable insights for potential therapeutic interventions and disease prevention. Several studies have shown that hematopoietic stem cells (HSCs) exhibited age-associated phenotypes as early as middle age [1]. However, HSCs are a heterogenous group of cells, and the question remains whether HSC subsets are differentially affected by aging. Our previous work demonstrated in young mice that leptin receptor (LEPR)^+^ HSCs, a highly engrafting subpopulation of HSCs expressing the truncated, non-functional isoform of LEPR under steady-state, exhibit transcriptomic programs enriched for type I interferon (IFN) and interferon-gamma (IFN-γ) associated pathways compared to LEPR^-^ HSCs [2]. Beyond its role in embryonic HSC development, it is known that chronic IFN-γ signaling leads to HSC cycling and ultimately exhaustion [3]. Similarly, while short-term type I interferon stimulation is critical during an acute infection, long-term exposure as in chronic inflammation compromises HSC repopulating function [4]. Therefore, we hypothesized that LEPR^+^ HSCs, due to their highly pro-inflammatory molecular programs at baseline, would be more highly susceptible to aged-associated dysfunctions.

**Fig. 1:**
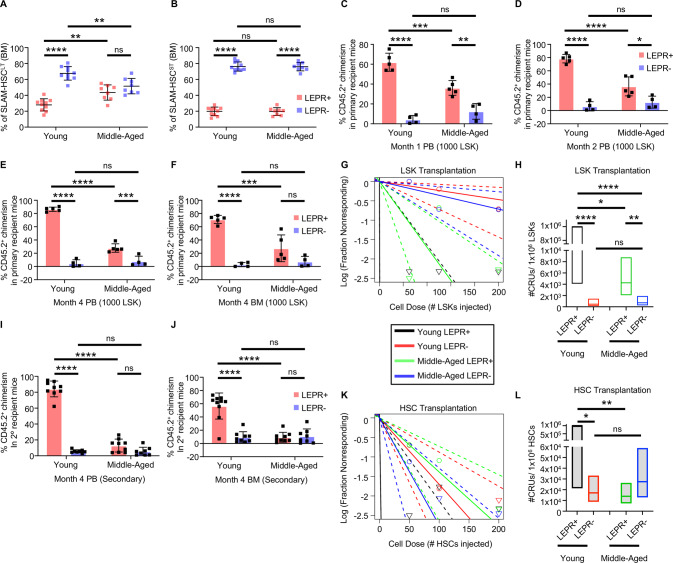
Long-term LEPR^+^ HSCs from middle-aged mice expanded in frequency with an accelerated decline in function as compared to young mice. **A**, **B** FACS analyses of freshly isolated total bone marrow (BM) cells from young and middle-aged C57BL/6 J (*n* = 8–10). Percentages of LEPR^+^ versus LEPR^-^ cells within SLAM-HSC^LT^ (LSK Flt3^-^CD150^+^CD48^-^) and SLAM-HSC^ST^ (LSK Flt3^-^CD150^-^CD48^-^), respectively. **C**–**F** Equal numbers of freshly sorted LEPR^+^LSK vs. LEPR^-^LSK cells from young or middle-aged C57BL/6 J femur BM were used at different doses (shown here dose 1000) for limiting dilution assay (LDA). Donor chimerism in peripheral blood (PB) month 1, 2, 4 and BM month 4, respectively. **G** Poisson statistical analysis from the LDA. Solid lines represent the best-fit linear model for each data set; dotted lines represent 95% confidence intervals. Symbols represent the percentages of negative mice for each cell dose. This plot has been modified from the original for clarity purpose. **H** No. of CRUs per one million transplanted cells calculated from (**G**); line representing median; box representing 95% confidence interval. **I**, **J** For secondary transplant, two millions of total BM cells pooled from primary hosts were intravenously injected into lethally irradiated secondary hosts. Donor chimerism in PB and BM at month 4, respectively. **K**, **L** Equal numbers of freshly sorted LEPR^+^ SLAM-HSCs (LSK CD150^+^CD48^-^) vs. LEPR^-^ SLAM HSCs from young or middle-aged C57BL/6 J femur BM were used at different doses for an LDA. **K** Poisson statistical analysis from the LDA. Solid lines represent the best-fit linear model for each data set; dotted lines represent 95% confidence intervals. Symbols represent the percentages of negative mice for each cell dose. This plot has been modified from the original for clarify purpose. **L** No. of CRUs per one million transplanted cells calculated from (**K**); line representing median; box representing 95% confidence interval. All data are mean ± SD. *p < 0.05, **p < 0.01, ***p < 0.001, ****p < 0.0001 using Ordinary Two-way ANOVA followed with post hoc Tukey’s multiple comparison test. *N* = 2 independently repeated experiments.

To test our hypothesis, we first assessed how aging altered phenotypically defined hematopoietic stem and progenitor cell proportions in the bone marrow (BM) of middle-aged (12–15-month-old) versus young (2–4-month-old) mice using fluorescence activated cell sorting (FACS) analysis. Consistent with our previous report, proportions of young LEPR-expressing long-term SLAM HSCs (Lin^-^Sca1^+^c-Kit^+^Flt3^-^CD150^+^CD48^-^) (LSK Flt3^-^CD150^+^CD48^-^, hereafter denoted as SLAM-HSC^LT^) and short-term SLAM-HSCs (LSK Flt3^-^CD150^-^CD48^-^ or SLAM-HSC^ST^) were significantly lower than LEPR^-^ counterparts (Fig. 1A, B) [2, 5]. However, LEPR^+^ SLAM-HSC^LT^ exist at a higher proportion in aged mice as compared to young mice (Fig. 1A). This same trend held true for a differently defined phenotypic LT-HSC (LSK CD34^-^Flt3^-^), ST-HSC (LSK Flt3^-^CD34^+^), multipotent progenitor (MPP, LSK Flt3^+^CD34^+^), and subsets of MPPs including myeloid-biased MPP2 (LSK Flt3^-^CD150^+^CD48^+^), myeloid-biased MPP3 (LSK Flt3^-^CD150^-^CD48^+^), and lymphoid-biased MPP4 (LSK Flt3^+^CD150^-^CD48^+^) (Fig. S1A-F) [6, 7]. Interestingly, the difference in proportions between LEPR^+^ and LEPR^-^ of the MPP2 subset in young mice essentially disappeared as the mice aged. Together, these data suggested that the expansion of LEPR-expressing subsets of phenotypic HSCs and MPPs occurred at different kinetics, and the populations were significantly altered as early as middle age. Alternatively, this could also be due to loss or differentiation of LEPR^-^ phenotypic HSCs and MPPs, or a combination of these factors.

HSCs from aged mice are present at higher numbers (Fig. S1G, H), but exhibit a significant decline in repopulating potential [8]. To determine whether middle-aged LEPR^+^ LSKs contained fewer competitive repopulating units (CRUs, a functional measure of murine HSCs) compared to the young cells, we performed limiting dilution engraftment assay (LDA) using increasing doses of equal numbers of LEPR^+^ or LEPR^-^ LSK cells from young or middle-aged mice. Consistent with previous studies, young LEPR^+^ LSK cells possessed superior repopulating capacity compared to LEPR^-^ LSK cells demonstrated by significantly higher donor chimerism in peripheral blood (PB) at month 1, 2, 4 (Fig. 1C–E) and BM at month 4 (Fig. 1F) as well as absolute number of CRUs (Fig. 1G, H, S2D) [2]. Compared to young LEPR^+^ LSK, middle-aged LEPR^+^ LSK cells showed significantly lower donor chimerism and lower number of CRUs. A similar trend was seen in the lower doses of injected cells (Fig. S2Ai-Biv). In contrast, middle-aged LEPR^- ^LSK cells showed no significant difference in engraftment or CRUs compared to young LEPR^-^ LSK cells. There were significant changes in percentage of lymphoid cells in peripheral blood of young LEPR^+^ compared to young LEPR^-^ HSC recipients, and there was a significant shift toward myeloid cells in bone marrow of recipients of aged HSC transplants regardless of LEPR status (Fig. S6A–D). To evaluate self-renewal capacity, we performed secondary transplant by injecting equal numbers of unseparated BM cells from primary hosts of each group into lethally irradiated secondary recipients. The chimerism results in PB at month 1–4 and BM at month 4 mirrored findings in primary transplant suggesting that compared to young LEPR^+^ HSCs, middle-aged LEPR^+^ HSCs not only exhibited engraftment dysfunction but also decreased self-renewing potential (Fig. 1I, J, S2Ci-ii). To directly compare changes in engraftment functions of young versus middle-aged LEPR-expressing HSCs, we performed another LDA using increasing doses of LEPR^+^ or LEPR^-^ SLAM-HSCs (LSK CD150^+^CD48^-^) cells from young or middle-aged mice. In line with the above transplant, middle-aged LEPR^+^ HSCs showed lowered chimerism in PB at month 1, 2, 4 and BM month 4 for all doses tested as compared to young LEPR^-^ HSCs (Fig. S3Ai-Civ). Consistent with that observation, the number of CRUs was significantly lower in middle-aged than young LEPR^+^ HSCs (Fig. 1K, L, S3D). In contrast, there was no significant difference in engraftment of middle-aged versus young LEPR^-^ HSCs demonstrated either as chimerism (Fig. S3Ai-Civ) or CRU quantification (Fig. 1K, L, S3D). Altogether, the data demonstrated that as the mice aged LEPR^+^ HSCs exhibited a decline in both engraftment and self-renewing capabilities that was not observed in LEPR^-^ HSCs.

**Fig. 2:**
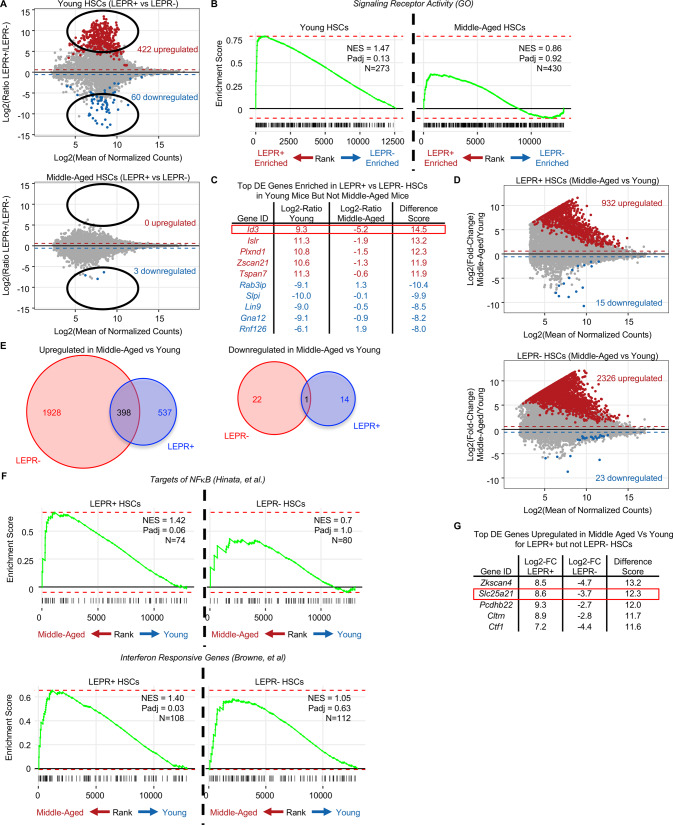
Aging induces heterogeneous transcriptomic responses in HSCs dependent on LEPR status. RNA-seq was performed on sorted viable young and middle aged LEPR^+^ and LEPR^-^ SLAM HSCs (LSK CD150^+^CD48^-^). **A** MA plot showing overview of changes in LEPR^+^ HSCs compared to LEPR^-^ HSCs from young (top) and middle-aged (bottom) mice. **B** Fast gene set enrichment showing enrichment of genes associated with signaling receptor activity in LEPR^+^ HSCs compared to LEPR^-^ HSCs for cells from young or middle-aged mice. **C** Top differentially expressed genes enriched in LEPR^+^ (red) or LEPR^-^ (blue) young HSCs but not in middle aged HSCs. **D** MA plot showing overview of changes in cells isolated from middle-aged vs young for LEPR^+^ (top) or LEPR- (bottom) HSCs. **E** Venn diagrams showing the overlap of genes upregulated (left) or downregulated (right) in cells from middle-aged vs young mice for LEPR^+^ or LEPR^-^ HSCs. **F** Fast gene set enrichment analysis showing enrichment of genes associated with NFkB signalling (top) and interferon response pathways (bottom) changed in middle aged compared to young for LEPR^+^ or LEPR^-^ HSCs. **G** Top differentially expressed genes upregulated in middle-aged vs young for LEPR^+^ HSCs but not LEPR^-^ HSCs. NES = normalized enrichment score; Padj = adjusted *p* value; N = number of genes in set; Log2-Ratio = log2(ratio of expression in young vs middle aged mice); Log2-FC = log2(fold-change of middle aged vs young).

Given the drastic decrease in repopulating potential of middle-aged LEPR^+^ HSCs compared to young cells, we performed RNA-sequencing on LEPR^+^ and LEPR^-^ SLAM HSCs (LSK CD150^+^CD48^-^) of young and middle-aged mice to characterize changes in their molecular signatures brought on by aging. As previously reported, young LEPR^+^ HSCs were molecularly distinct from LEPR^-^ HSCs (Fig. 2A, Table S1) [2]; however, there were only 3 genes downregulated in middle-aged LEPR^+^ compared to LEPR^-^ HSCs (Fig. 2A, S4A). In young mice, LEPR^+^ HSCs were significantly enriched for genes associated with signaling receptor activity compared to LEPR^-^ HSCs; this was not observed in middle-aged HSCs (Fig. 2B), which could contribute to the change in function of middle-aged compared to young LEPR^+^ HSCs. Furthermore, young but not middle-aged LEPR^-^ HSCs were highly enriched for myeloid cell migration compared to LEPR^+^ HSCs (Fig. S4B). Among the top genes enriched in young LEPR^+^ HSCs but not middle-aged mice is *Id3* (Fig. 2C). Interestingly, both *ID1* and *ID3* were reportedly upregulated upon hematopoietic induction of human pluripotent stem cell [9] and were required for steady-state hematopoiesis by maintaining endothelial cell integrity [10].

Consistent with previous studies that HSCs show an aged phenotype as early as middle-age, our data revealed that both subsets of HSCs regardless of LEPR status had significantly different gene profiles compared to their corresponding young cells (Fig. 2D, E) [1, 8]. Both middle-aged LEPR^+^ HSCs and LEPR^-^ HSCs were significantly enriched for gene programs linked to aging including increased expression of PRC2 targets (Fig. S5Ai) and decreased expression of genes associated with oxidative phosphorylation, such as *Lgals1* (Fig. S5Aii, C) [11]. Of note, among genes upregulated in middle-aged HSCs was *Ncam1*, which was found in several AML subgroups and linked to drug resistance in AML, a disease well-known to be associated with age and clonal hematopoiesis (Fig. S5B) [12, 13]. However, there were also gene pathways that distinguished LEPR^+^ from LEPR^-^ HSCs as the mice age. Specifically, compared to young mice, middle-aged LEPR^+^ HSCs were significantly enriched for proinflammatory pathways including *NFκB* target genes and interferon responsive genes (Fig. 2F) as well as hematopoietic progenitor cell differentiation (Fig. S5D); these were not observed in LEPR^-^ HSCs. This suggested that the proinflammatory transcriptomic profile in young LEPR^+^ HSCs were further exacerbated in middle-aged HSCs, which acted as an important factor contributing to the early age-associated decline in function of LEPR^+^ HSCs but not LEPR^-^ HSCs. In addition, differential expression analysis showed many genes altered in middle-aged versus young cells in LEPR^+^ HSCs that were unchanged in LEPR^-^ HSCs. This included genes associated with normal hematopoietic development, such as *Sparc*, that were more strongly downregulated by aging in LEPR^+^ HSCs than in LEPR^-^ HSCs (Fig. S5E) [14], and genes associated with aging phenotypes such as mitochondrial dysfunction like *Slc25a21*, a mitochondrial transport gene (Fig. 2G) [15]. Taken together, this transcriptomic data reinforced the idea that mouse HSCs age heterogeneously and showed that LEPR^+^ HSCs are profoundly affected by age.

In summary, we report in this study that the effect of aging on subsets of murine BM HSCs was most dramatic in a subset of cells. Specifically, LEPR-expressing HSCs while possessing remarkable repopulating potential and self-renewal capacity in young mice were demonstrated here with significant characteristics of age-associated phenotypes including increased frequency, diminished functions and exacerbated pro-inflammatory transcriptional programs beginning in middle-age range as compared to the rest of HSCs. However, middle-aged LEPR^-^ HSCs did not show significant decline in engraftment or self-renewing properties. Hence, our work suggested that LEPR-expressing HSCs are a potential target for therapies to reverse or delay the detrimental impact of aging in the blood. It is well-appreciated that aging is associated with increased incidence of clonal hematopoiesis, leukemic transformation and hematological disorders, and this process starts with the HSC, the cell that gives rise to all other blood and immune cells [13]. Hence, it is very important to delineate precisely how different subsets of HSCs undergo aging. In addition, aging is also associated with alterations in metabolism, so factors like weight, diets or sex could all potentially affect age-associated phenotypes. Future studies are warranted to determine the potential intrinsic and extrinsic factors, including those produced by the bone marrow niche, that govern these changes in LEPR-expressing HSCs.

## Supplementary information

Supplementary Figures

Supplementary Table

## Data Availability

RNA-seq raw and processed data files are available in the Gene Expression Omnibus (GEO GSE221126). All other data will be available on request.
