# Supplementary material for: Age-related decline in LEPR+ hematopoietic stem cell function
Source: Leukemia. 2023 Jan 17;37(3):712–6. doi: 10.1038/s41375-023-01815-1 (PMC9991916; doi:10.1038/s41375-023-01815-1)
Supplement: Supplementary file 1 — Supplementary Figures [file 41375_2023_1815_MOESM1_ESM.pdf]

Supplemental material and methods

Mice

Young (2-3 months old) and middle-aged (12-15 months old) female and male C57BL/6J (CD45.1−CD45.2+), Boy/J (CD45.1+CD45.2−) (2-3 months old), and C57BL/6JxBoy/J F1 (CD45.1+CD45.2+) (herein referred to as F1) were supplied in house by the In Vivo Therapeutics Core at Indiana University School of Medicine. Mice were housed in groups of 5 according to age, sex, and genotype under light and temperature-controlled conditions (12 h light/12 h dark cycle, 21–24 ÅãC).

Cell harvest, enrichment of mouse Lineage negative (Lin-) bone marrow (BM) cells, flow cytometry analysis and sorting

BM was obtained by flushing mouse femurs with ice-cold MACS buffer (PBS supplemented with 0.5% BSA and 2 mM EDTA) and broken up into single cell suspension by pipetting with Eppendorf Combitips (Eppendorf). For RNA-seq sample preparation, BM was harvested by crushing spines, pelvis, femurs and tibias in ice-cold MACS buffer, and lineage depletion was done per manufacturer's protocol of the direct mouse lineage cell depletion kit (Miltenyi Biotec).

For staining of LEPR, BM cells were first incubated with anti-LEPR biotin-conjugated antibody (R&D systems, BAF497, 0.2 µg per one million cells) and fluorescently conjugated primary anti-mouse antibodies (0.2 µg of antibody was used per one million cells) for 20 min, washed in MACS buffer, then incubated with secondary streptavidin-PE/Cy7 on ice (Biolegend, 1:500) for 20 min and washed in MACS buffer. Cells were resuspended in DAPI solution (BD Biosciences, 1:10,000) and analyzed using LSR II flow cytometer (BD Biosciences) or sorted using a FACSAria or SORP Aria flow cytometers (BD Biosciences). For analyses of transplants, BM cells were counted and stained at a concentration of 2.5–4 Å~ 106 cells in 200 µl MACS buffer per tube on ice for 20 min and washed in MACS buffer.

The following conjugated primary anti-mouse antibodies were used for staining LEPR on BM hematopoietic and progenitor cells: APC- (BD Biosciences) or FITC-mouse lineage cocktail (CD3e, CD11b, CD45R/B220, Ly-76, Ly-6G and Ly-6C; Biolegend), PE-CF594 anti-Ly-6A/E (clone D7, BD Biosciences) or PE/Dazzle 594 anti-Ly6A/E (also known as Sca-1; clone D7; Biolegend), APC/Cyanine7 anti-CD117 (also known as c-kit; clone 2B8; Biolegend), Brilliant Violet BV421 rat anti-CD150 (clone Q38-480, BD Biosciences) or PerCP/Cyanine5.5 anti-CD150 (clone TC15-12F12.2, Biolegend), FITC anti-CD48 (clone HM48-1; Biolegend), PE anti-CD34 (clone MEC14.7, Biolegend), APC- (clone A2F10) or PE anti-CD135 (also known as Flt3; clone A2F10; Biolegend).

For transplantation assays, the following conjugated primary anti-mouse antibodies purchased from Biolegend were used: APC anti-CD45.2 (clone 104), FITC anti-CD45.1 (clone A20), Brilliant Violet BV421 anti-CD3 (clone 17A2), PE/Cy7 anti-CD11b (M1/70), and PerCP/Cyanine5.5 anti-CD45R/B220 (clone RA3-6B2).

FlowJo 10.7 software (BD) was used for data analysis. Compensation using single-colored controls was done for each experiment; gating was based on fluorescence minus one controls.

Limiting dilution analysis

Freshly sorted LEPR+ LSK and LEPR- LSK of increasing doses (250, 500, 1000) or LEPR+ HSC (LSK CD150+ CD48-) and LEPR- HSC of increasing doses (50, 100, 200) from pooled BM of C57BL/6J (CD45.1−CD45.2+) donor were i.v. injected with 50,000 or 100,000 respectively of unseparated BoyJ (CD45.1+CD45.2−) BM cells into F1 recipient (CD45.1+CD45.2+) mice (n = 5–7) that had been lethally irradiated (700 cGy followed with 400 cGy) 24 h prior to transplantation. Donor chimerism (%CD45.1−CD45.2+) was determined in PB at month 1, 2, 4 and BM at month 4. The number of mice with 2% (LEPR+ LSK vs. LEPR LSK transplant) or 1% (LEPR+ HSC vs. LEPR- HSC transplant) or greater of donor-derived BM was assessed for each dose. Frequencies of competitive repopulating units (CRUs) were calculated and plotted using ELDA software (http://bioinf.wehi.edu.au/software/elda/) and the "limdil" package in R.

RNA-seq methods

RNA was harvested from sorted viable LEPR+ or LEPR-HSCs isolated from young or middle-aged mice using Qiagen RNeasy plus micro kit. Libraries were prepared using NEBNext Ultra Low/ Single Cell RNA sequencing library prep kit. Libraries were pooled and sequenced on an Illumina NovaSeq S2 flowcell to obtain paired end 100bp reads. Quality of reads were analyzed using FastQC, and reads were trimmed of adapters and quality trimmed using Cutadapt with the following options: -a AGATCGGAAGAGCACACGTC -A AGATCGGAAGAGCGTCGTGT --nextseq-trim=20 --minimum-length 50. Genome indices were generated using the GRCm39 mouse genome downloaded from GENCODE (Release M27) using STAR sequence aligner with the following options: --sjdbOverhang 99. Reads were aligned using STAR sequence aligner with the following options: --readFilesCommand zcat –outSAMtype BAM SortedByCoordinate --outSAMunmapped Within. Reads were counted using HTSeq assigning reads to genes using the following options: -s no -r pos -f bam. DESeq2 R package was used to perform differential gene expression analysis using the design ~Mouse + LEPRstatus for comparison of LEPR+ to LEPR- within age groups and the design ~Sex + Age for comparisons within the same LEPR status crossing age groups. Cutoff for DE genes were alpha=0.05, lfcThreshold=log2(1.5). The R package "fgsea" was used to perform gene set analysis using the MSigDB curated datasets C2, C5, and C6 that have been mapped to mouse orthologs and are publicly available from https://bioinf.wehi.edu.au/software/MSigDB/. Statistics were calculated for fgsea analysis using the shrunken log2FoldChange values from DESeq2 using the apeglm lfcShrink parameter. Fgsea was performed using the parameters minSize=50, maxSize=500, nPermSimple=100000. RNA-seq raw and processed data files will be deposited to the Gene Expression Omnibus (GEO).

Statistical analyses

Results are shown as mean values Å} SD. A p value < 0.05 was considered statistically significant. Two-way ANOVA with post hoc Tukey's multiple-comparisons test was used. For limiting dilution assay transplants, Poisson statistical analysis was used. Graphpad Prism outlier calculator (alpha = 0.05) was used to exclude outlier data points (https://www.graphpad.com/quickcalcs/agrubbs1/).

Supplemental Tables and Figures

Supplemental Table 1 - .xlsx file with the differential expression analyses for all comparisons discussed in this study.

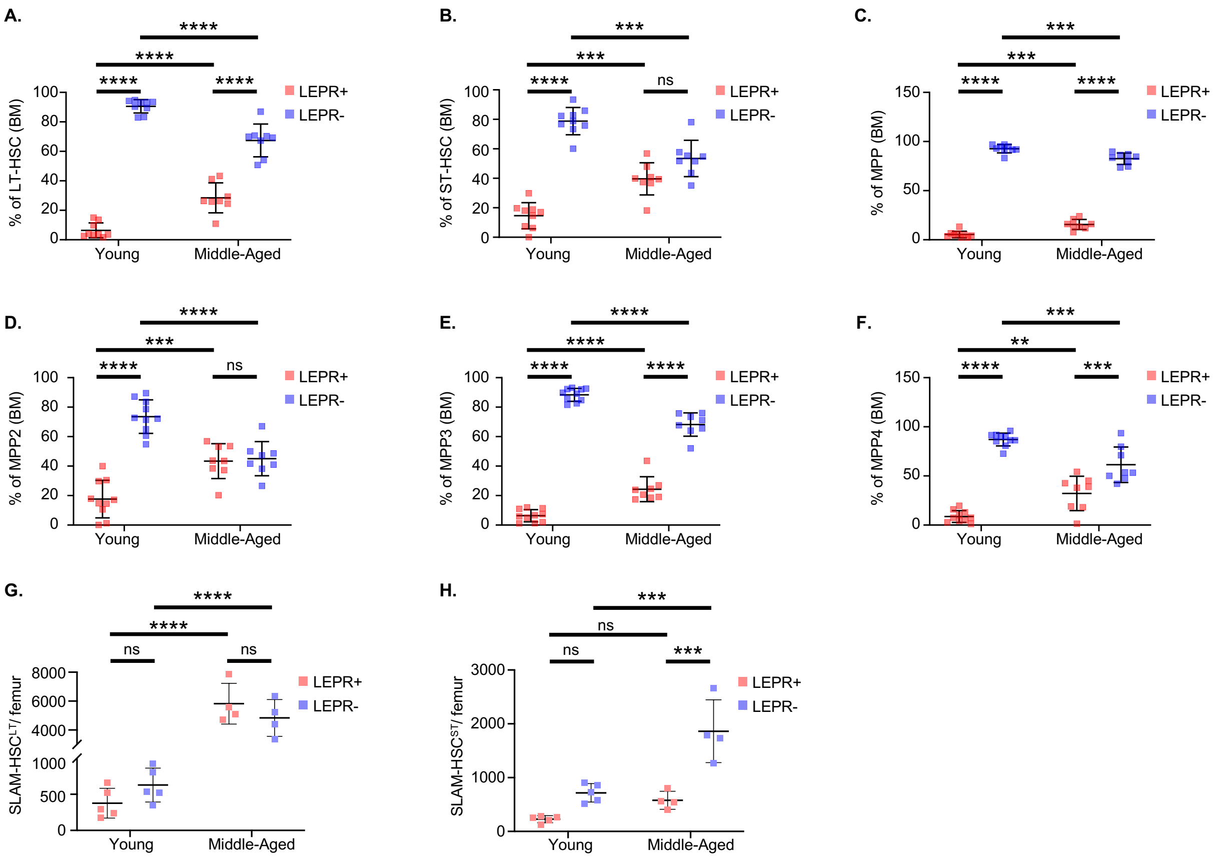

Supplemental Figure 1 – LEPR-expressing bone marrow (BM) stem and progenitor cells from middle-aged mice expanded in frequency and absolute numbers as compared to young mice. FACS analyses of freshly isolated total BM cells from young and middle-aged C57BL/6J (n=8-10). A-F. Percentages of LEPR+ versus LEPR- cells within phenotypic LT-HSC (LSK CD34-Flt3-), ST-HSC (LSK Flt3-CD34+), multipotent progenitor (MPP, LSK Flt3+CD34+), MPP2 (LSK Flt3-CD150+CD48+), MPP3 (LSK Flt3-CD150-CD48+) and MPP4 (LSK Flt3+CD150- CD48+), respectively. G-H. Absolute numbers per femur of LT- and ST-HSCs based on LEPR status in young and middle aged mice (n=4-5). 2-way ANOVA with post hoc Tukey test: *p<0.05; **p<0.01; ***p<0.001; ****p<0.0001.

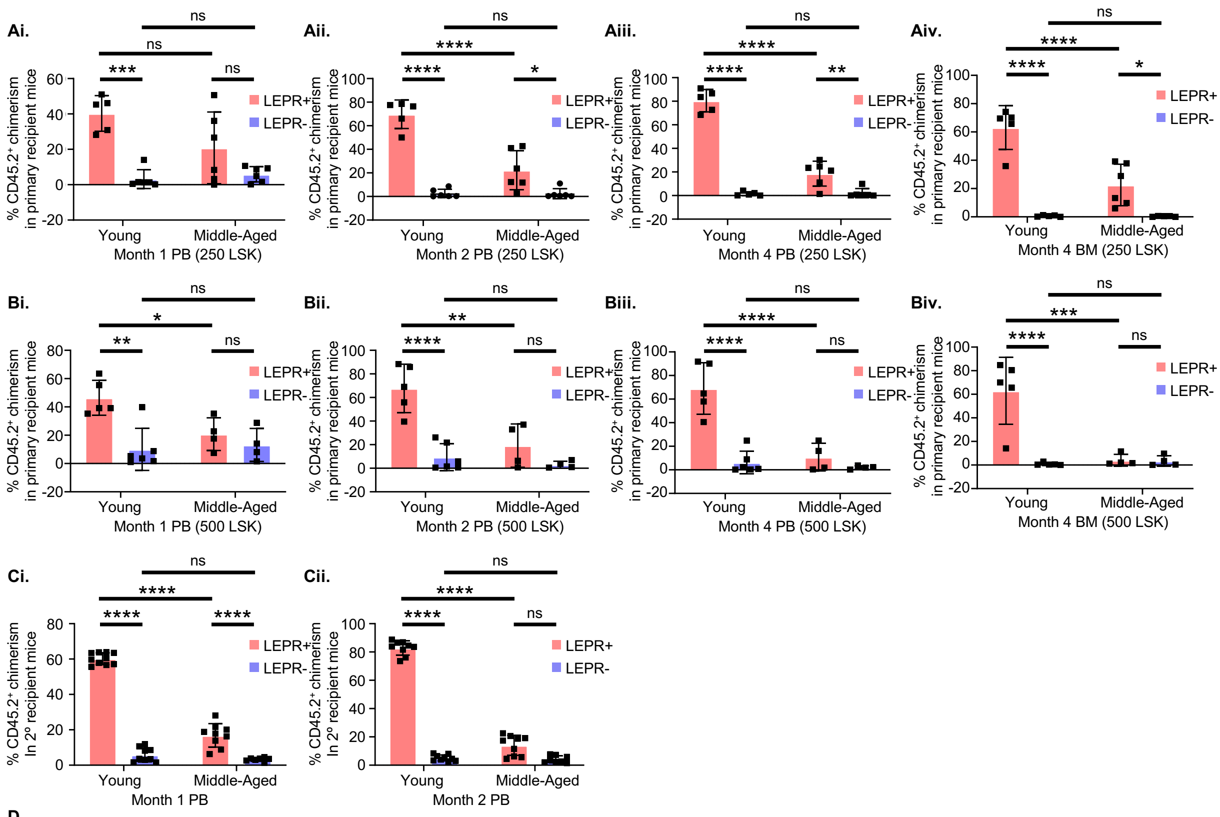

D

Data Used for Calculating Competitve Repopulating Frequencies (Input into ELDA Software)				Calculated Competitive Repopulating Frequencies		
**Group**	**Dose**	**Number in Group**	**Number Showing Engraftment**	**Estimate**	**High**	**Low**
**Young LEPR+ LSK**	250	5	5			
**Young LEPR+ LSK**	500	5	5	**1/1**	1/1	1/244
**Young LEPR+ LSK**	1000	5	5			
**Young LEPR- LSK**	250	5	0			
**Young LEPR- LSK**	500	5	1	**1/2138**	1/697	1/6558
**Young LEPR- LSK**	1000	4	2			
**Middle-Aged LEPR+ LSK**	250	6	6			
**Middle-Aged LEPR+ LSK**	500	4	2	**1/235**	1/114	1/485
**Middle-Aged LEPR+ LSK**	1000	5	5			
**Middle-Aged LEPR- LSK**	250	5	0			
**Middle-Aged LEPR- LSK**	500	4	2	**1/1401**	1/523	1/3750
**Middle-Aged LEPR- LSK**	1000	4	2			

The Likelihood ratio test of the single-hit model was not rejected (χ^2 = 1.135, p = 0.713) and the Score test of heterogeneity was not rejected (χ^2 = 7.9e-4, p = 0.978), thus the single hit model used in ELDA was appropriate.

Supplemental Figure 2 - LEPR+ LSK cells exhibited significantly higher engraftment capability and selfrenewing capacity as compared to LEPR- LSK cells in young mice but showed a significant functional decline in middle-aged mice. For primary transplant, freshly sorted LEPR+ LSK and LEPR- LSK cells of increasing doses (250, 500, 1000) from pooled flushed BM of C57BL/6J (CD45.1−CD45.2+) donor were i.v. injected along with 50,000 unseparated BoyJ (CD45.1+CD45.2−) BM cells into lethally irradiated (700 cGy followed with 400 cGy) F1 recipient (CD45.1+CD45.2+) mice (n = 5–7). Ai-iv. Donor chimerism was determined in PB at month 1, 2, 4 and BM at month 4 of dose 250, respectively. Bi-iv. Donor chimerism was determined in PB at month 1, 2, 4 and BM month 4 of dose 500, respectively. For secondary transplant, 2 millions of total BM cells which were collected by flushing and pooling from primary recipients of dose 1000 were i.v. injected into lethally irradiated secondary recipients. Ci-ii. Donor chimerism was determined in PB at month 1 and 2, respectively. D. For each group and dose, the numbers of primary recipients with a cut-off value (of engraftment in the bone marrow at month 4) of at

least 2% out of total number of recipients transplanted were used to calculate competitive repopulating units (CRUs). 2-way ANOVA with post hoc Tukey test: *p<0.05; **p<0.01; ***p<0.001; ****p<0.0001.

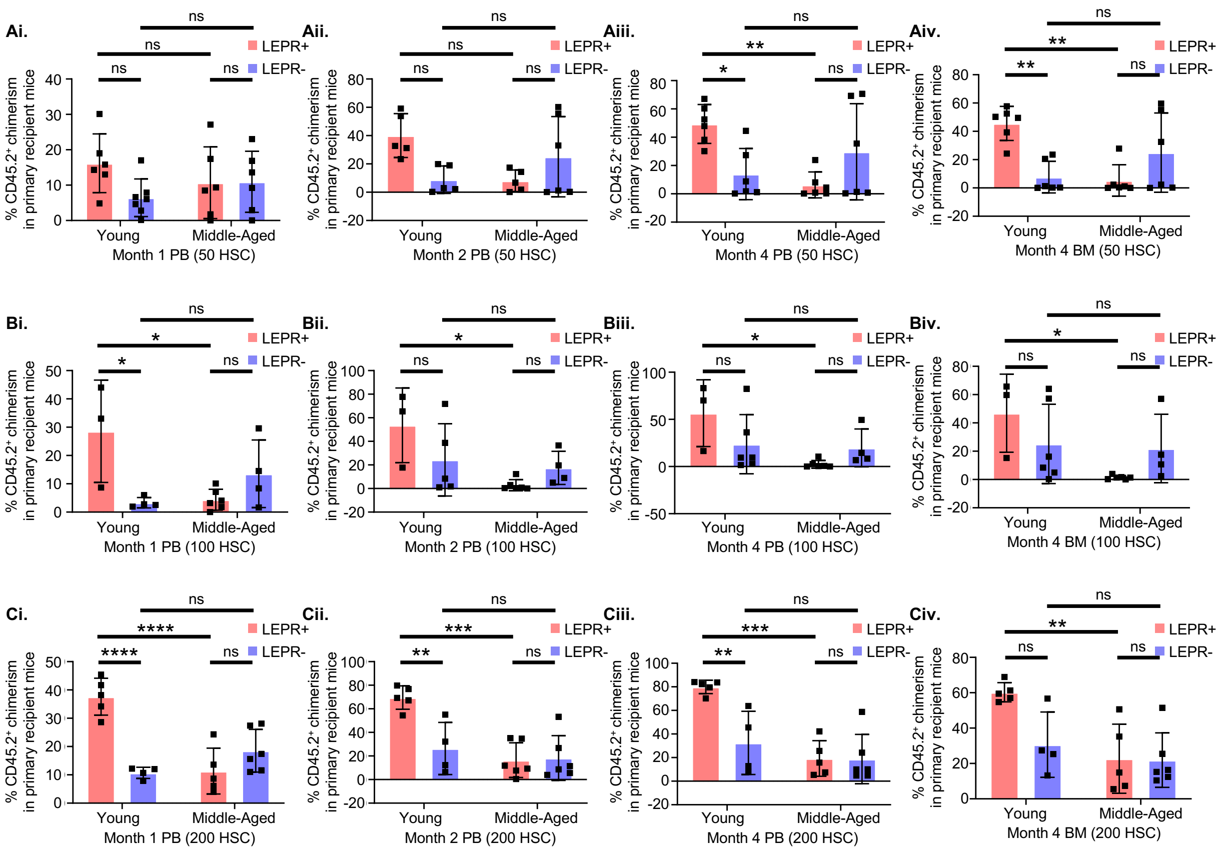

D

Data Used for Calculating Competitve Repopulating Frequencies (Input into ELDA Software)				Calculated Competitive Repopulating Frequencies		
Group	Dose	Number in Group	Number Showing Engraftment	Estimate	High	Low
Young LEPR+ HSC	50	6	6	**1/1**	1/1	1/47
Young LEPR+ HSC	100	3	3			
Young LEPR+ HSC	200	5	5			
Young LEPR- HSC	50	6	3	**1/58**	1/30	1/114
Young LEPR- HSC	100	6	5			
Young LEPR- HSC	200	4	4			
Middle-Aged LEPR+ HSC	50	6	3	**1/72**	1/37	1/137
Middle-Aged LEPR+ HSC	100	6	4			
Middle-Aged LEPR+ HSC	200	5	5			
Middle-Aged LEPR- HSC	50	6	4	**1/36**	1/17	1/77
Middle-Aged LEPR- HSC	100	4	4			
Middle-Aged LEPR- HSC	200	6	6			

The Likelihood ratio test of the single-hit model was not rejected (χ^2 = 0.97, p = 0.323) and the Score test of heterogeneity was not rejected (χ^2 = 0.749, p = 0.387), thus the single hit model used in ELDA was appropriate.

Supplemental Figure 3 - LEPR+ HSCs possessed significantly higher repopulating potential in young mice as compared to LEPR- HSCs but exhibited functional impairment in middle-aged mice. For primary transplant, freshly sorted LEPR+ HSCs and LEPR- HSCs of increasing doses (50, 100, 200) from pooled flushed BM of C57BL/6J (CD45.1−CD45.2+) donor were i.v. injected along with 100,000 unseparated BoyJ (CD45.1+CD45.2−) BM cells into lethally irradiated (700 cGy followed with 400 cGy) F1 recipient (CD45.1+CD45.2+) mice (n = 5–7). Ai-iv. Donor chimerism was determined in PB at month 1, 2, 4 and BM at month 4 of dose 50, respectively. Bi-iv. Donor chimerism was determined in PB at month 1, 2, 4 and BM at month 4 of dose 100, respectively. Ci-iv. Donor chimerism was determined in PB at month 1, 2, 4 and BM at month 4 of dose 200, respectively. D. For each group and dose, the numbers of primary recipients with a cut-off value (of engraftment in the bone marrow at month 4) of at least 2% out of

total number of recipients transplanted were used to calculate competitive repopulating units (CRUs). 2-way ANOVA with post hoc Tukey test: *$p<0.05$; **$p<0.01$; ***$p<0.001$; ****$p<0.0001$.

A

DE Genes Enriched in LEPR- vs LEPR+ HSCs
in Middle-Aged But Not Young Mice

Gene ID	Log2-Ratio Young	Log2-Ratio Middle-Aged
Sh2b2	1.4	-7.1
Zfp354a	0.3	-7.9
Jmjd4	0.5	-6.3

B

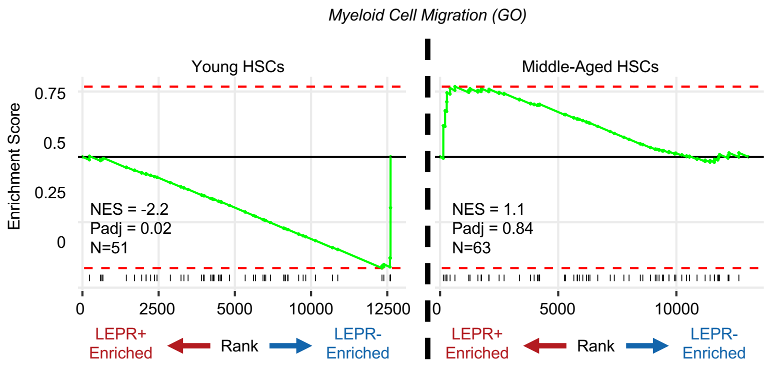

Supplemental Figure 4 – LEPR- HSCs were significantly enriched for genes associated with myeloid cell migration in young but not middle-aged mice as compared to LEPR+ HSCs. A. Differential expression of genes in LEPR- vs. LEPR+ HSCs (LSK CD150+CD48-) in middle-aged mice but not young mice. B. Gene set enrichment analysis of young vs. middle-aged HSCs against LEPR status. NES = normalized enrichment score; Padj = adjusted p-value; N = number of genes in set; Log2-FC = log2(fold-change of LEPR+ vs LEPR-).

Ai

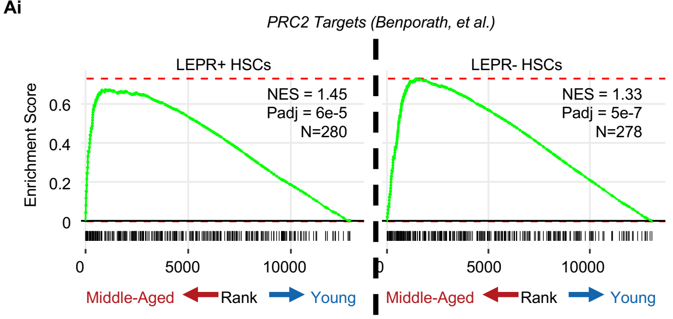

B

Top DE Genes Upregulated in Middle Aged Vs Young for both LEPR+ and LEPR- HSCs

Gene ID	Log2-FC LEPR+	Log2-FC LEPR-	Combined Score
Fbxo2	22.9	8.9	31.8
Map1b	9.9	12.1	22.0
Myo1d	10.2	11.6	21.8
Ncam1	10.0	11.6	21.6
Pde9a	9.3	11.8	21.0

Aii

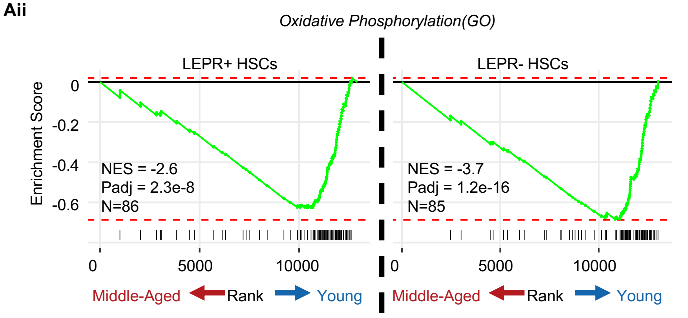

C

DE Gene Downregulated in Middle Aged Vs Young for both LEPR+ and LEPR- HSCs

Gene ID	Log2-FC LEPR+	Log2-FC LEPR-	Combined Score
Lgals1	-2.3	-1.6	-3.9

D

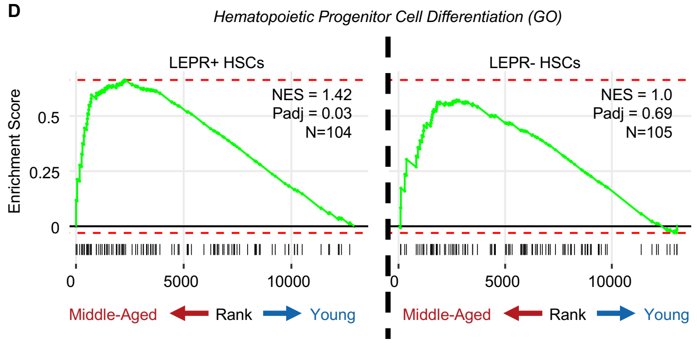

E

Top DE Genes Downregulated in Middle Aged Vs Young for LEPR+ but not LEPR- HSCs

Gene ID	Log2-FC LEPR+	Log2-FC LEPR-	Difference Score
Lrg1	-10.7	3.1	-13.8
Plvap	-8.9	3.1	-12.0
Sparc	-7.8	-0.2	-7.5
Ushbp1	-6.5	0.6	-7.2
5730409E04Rik	-3.3	2.5	-5.8

Supplemental Figure 5 – HSCs exhibited aging-associated transcriptomic phenotype regardless of LEPR status but only LEPR+ HSCs were significantly enriched for genes associated with hematopoietic progenitor cell differentiation in middle-aged mice. Ai-ii. Gene set enrichment analyses in LEPR+ vs. LEPR- in young compared to middle-aged mice. B-E. Differential expression of genes in middle-aged vs. young HSCs against LEPR status. NES = normalized enrichment score; Padj = adjusted p-value; N = number of genes in set; Log2-FC = log2(fold-change of middle aged vs young).

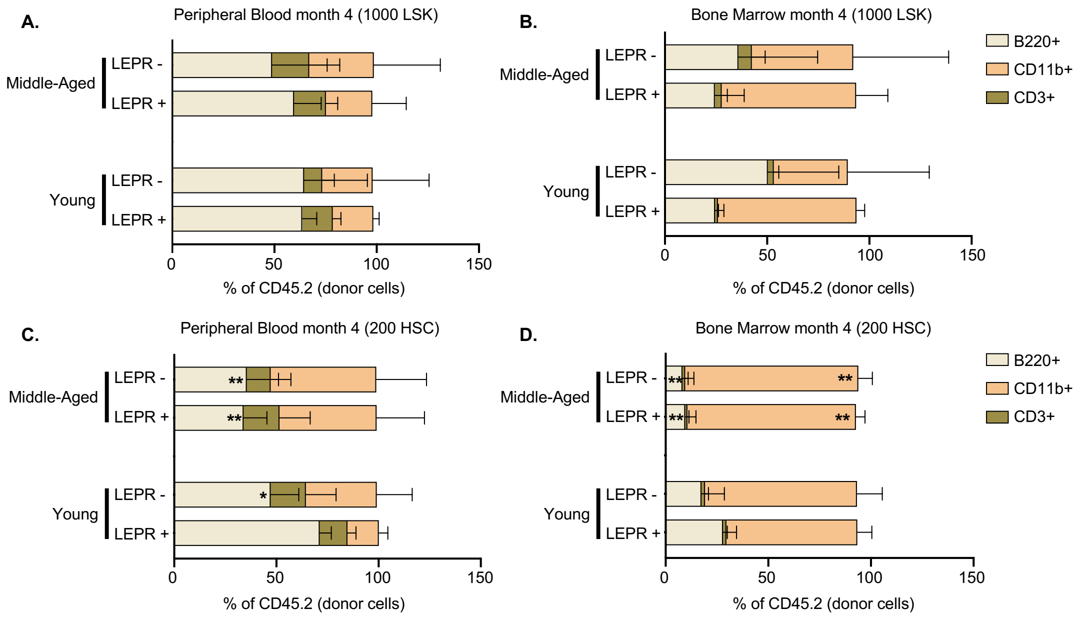

Supplemental Figure 6 – A-D. Percentage of B-cells (B220+), T-cells (CD3+), and myeloid cells (CD11b+) were calculated as a fraction of CD45.2+ donor cells in the peripheral blood (A/C) or bone marrow (B/D) of recipient mice receiving 1000 LSK (A-B) or 200 HSC (C-D) 4 months after transplantation to lethally irradiated recipients. 2-way ANOVA with Tukey test (significance shown for comparisons to Young LEPR+ groups): *p<0.05; **p<0.01.
